# Esthetic Reconstruction of Severe Vertical Soft Tissue Deficiency in the Lateral Incisor Region

**DOI:** 10.1002/ccr3.73063

**Published:** 2026-06-28

**Authors:** Ghassan Habash, Ayah Thikrallah, Mudar S. Kamal, Soher Nagi Jayash

**Affiliations:** ^1^ Department of Dental Sciences, Faculty of Graduate Studies Arab American University Ramallah Palestine; ^2^ University of Health Science Lahor Pakistan; ^3^ Palestinian Dental Association Al‐Quds Palestine; ^4^ Department of Fixed and Removal Prosthodontics, School of Dentistry Al‐Quds University Al‐Quds Palestine; ^5^ The Roslin Institute, College of Medicine and Veterinary Medicine University of Edinburgh Edinburgh UK

**Keywords:** 3D soft tissue augmentation, papillary preservation flap, photobiostimulation, vertical soft tissue deficiency

## Abstract

This case report describes management of a severely resorbed ectopic maxillary lateral incisor using atraumatic extraction, socket preservation with coral graft, and vertical soft tissue augmentation via a rotational pedicle connective tissue graft. A resin‐bonded bridge restored the site, achieving stable soft tissues and satisfactory long‐term esthetic and functional outcomes.

## Key Clinical Message

Predictable minimally invasive surgical‐prosthetic approach preserves pontic site and soft tissue volume after anterior tooth loss when implant therapy is contraindicated.

**Abbreviations**
L‐PRF: autologous leukocyte platelet rich fibrin
OPG: orthopantomogram
PBST: photobiostimulation therapy

## 1 Introduction

The loss of a maxillary lateral incisor, particularly in the esthetic zone, is often associated with rapid dimensional changes of the alveolar ridge and soft tissue collapse, which may compromise subsequent prosthetic outcomes [1]. Preservation of hard and soft tissues following tooth extraction in the anterior maxilla remains a critical challenge in contemporary dental practice. When such teeth are located in the esthetic zone, careful treatment planning is required to maintain papillary architecture, gingival contour, and ridge volume. Immediate or early intervention aimed at preserving the extraction site is essential, particularly when implant therapy is contraindicated, deferred, or declined [2, 3]. Alveolar ridge preservation techniques using bone graft materials have been widely advocated to minimize post‐extraction ridge resorption [4]. However, preservation of hard tissue alone is often insufficient to achieve optimal esthetic outcomes, especially for pontic site development where adequate vertical and horizontal soft tissue volume is paramount. Various soft tissue augmentation techniques have been described to enhance pontic site contours, including connective tissue grafts and pedicle flaps, each with specific indications and limitations [5, 6]. Rotational pedicle flaps harvested from the palatal mucosa offer the advantage of maintaining vascular supply while increasing soft tissue volume and vertical dimension at deficient sites [7]. When combined with atraumatic extraction, papilla‐preserving flap design, and socket grafting, such techniques may contribute to favorable esthetic and functional outcomes in the anterior maxilla. This case report describes the interdisciplinary management of an ectopically positioned maxillary lateral incisor affected by severe cervical resorption. The treatment approach involved atraumatic extraction, socket preservation using a natural coral‐derived bone graft, vertical soft tissue augmentation with a rotational palatal pedicle flap, and final restoration with a resin‐bonded prosthesis retained by labial veneers. The objective of this report is to highlight a predictable technique for achieving adequate soft tissue volume and esthetic pontic site development in challenging anterior maxillary cases.

## 2 Case History

**FIGURE 1:**
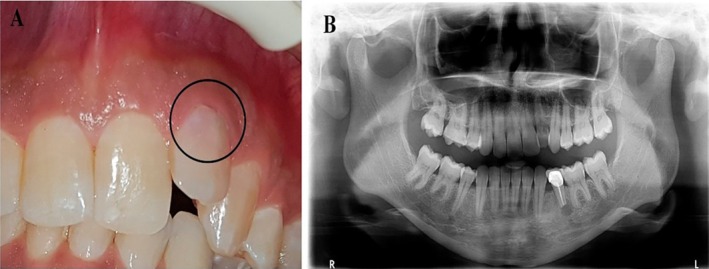
(A) Clinical photograph shows pink color at the cervical portion of the upper left lateral incisor. (B) Orthopantomogram showing an external root resorption of the maxillary left lateral incisor.

A 34‐year‐old, medically free female patient attended a private periodontology clinic seeking to treat a lower left premolar tooth with extraction and implant. Comprehensive clinical examinations, including intraoral and extraoral assessments, were performed. A buccal pink color was noticed at the cervical portion of the upper left lateral incisor (Figure 1A).

## 3 Therapeutic Intervention

**FIGURE 2:**
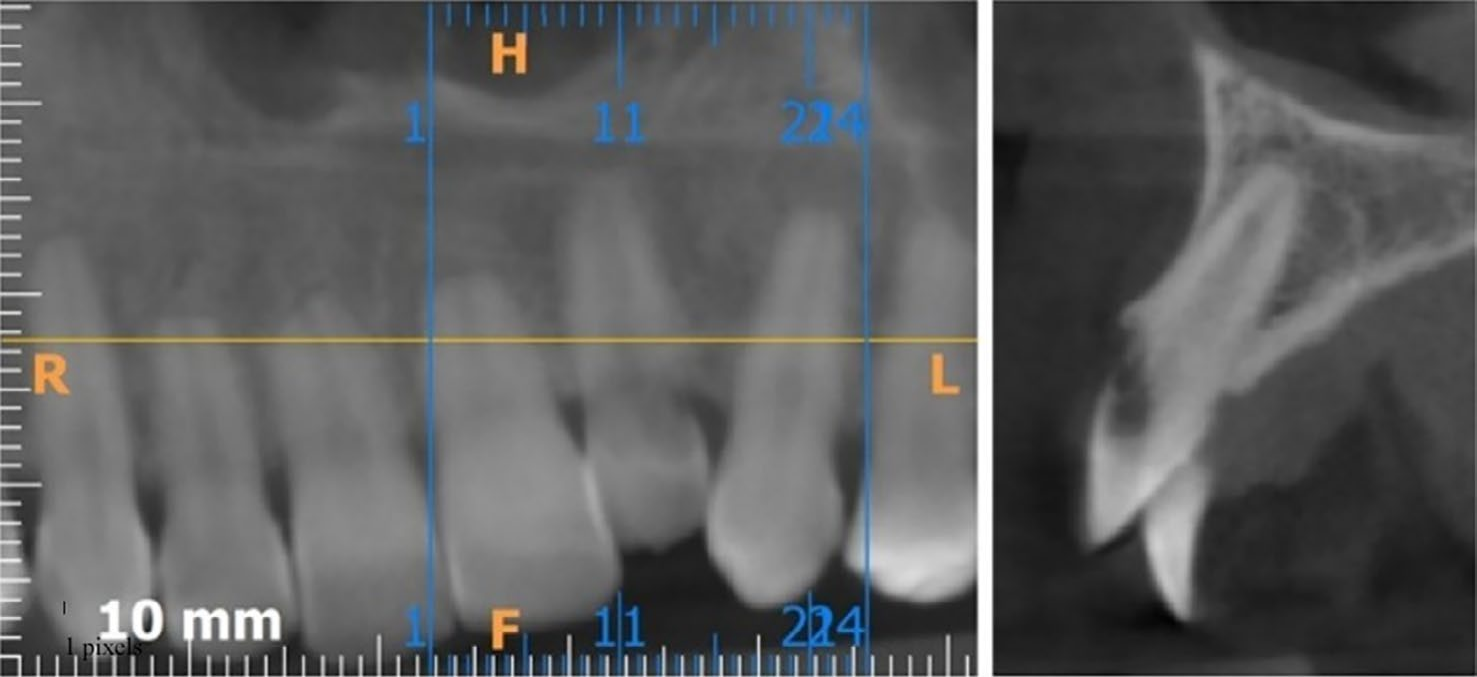
CBCT imaging confirmed cervical resorption of the upper left lateral incisor.

An orthopantomogram (OPG) revealed that a massive cervical root resorption with minimal sound tooth structure was noticed (Figure 1B). CBCT scan was ordered to obtain a comprehensive assessment of the tooth's position and surrounding structures (Figure 2).

After careful analysis of OPG and CBCT, a digital study model was fabricated and a virtual extraction of the lateral incisor was done to observe the defect resulting from and accordingly multiple treatment options discussed.

Multiple treatment options were discussed from periodontal, orthodontic, and prosthodontic perspectives. Orthodontic extrusion to reposition the tooth along with the surrounding hard and soft tissues was declined due to the severely shortened, resorbed root resulting from a previous prolonged orthodontic treatment period of approximately 5 years.

Placing an implant immediately after extraction will most likely result in an esthetically unsightly prosthetic result; where the crown length is disproportionate with its width and a zenith point that is significantly apical to the adjacent natural teeth. This was an expected consequence since bone grafting alone will not compensate for the vertical discrepancy and the expected loss of the thin labial cortical plate.

Maintaining the papilla height poses a greater challenge. According to Salama et al. [8] classification Table 1. The best probability to have an ideal interdental papilla is to have 6.5 mm tooth pontic distance. The reduced mesio‐distal space of the extraction site and the malaligned adjacent teeth (central incisor and canine) pose additional difficulties complicating the prosthodontic replacement of the lateral incisor.

**TABLE 1:** Salama et al. classification of predicted interdental papilla height.

Class	Restorative environment	Proximity limitations (mm)	Vertical soft tissue limitations (mm)
1	Tooth–tooth	1.0	5.0
2	Tooth–pontic	N/A	6.5
3	Pontic–pontic	N/A	6.0
4	Tooth–implant	1.5	4.5
5	Implant–pontic	N/A	5.5
6	Implant–implant	3.0	3.5

Another option involved extraction of the tooth followed by vertical soft tissue grafting and restoration with a conventional fixed bridge; however, this approach was considered overly invasive, particularly taking into consideration the malaligned central incisors and the slightly tipped canine. Consequently, a resin‐bonded bridge with two labial veneers was proposed. The veneers would retain the prosthetic lateral incisor and would offer a conservative solution to align the centrals and canines. The following is the timeline of clinical events (Table 2).

**TABLE 2:** Timeline of clinical events.

Time	Event
Initial visit	Patient presented with ectopically positioned maxillary lateral incisor with severe cervical resorption
Diagnosis	Clinical and radiographic assessment confirmed non‐restorable tooth
Surgery	Atraumatic extraction, papillary preservation flap, and socket preservation using coral bone graft
Soft tissue augmentation	Rotational pedicle connective tissue graft with de‐epithelialized connective tissue punches
Prosthetic phase	Resin‐bonded bridge retained by two labial veneers
Follow‐up	Stable soft tissue volume and satisfactory esthetic outcome

**FIGURE 3:**
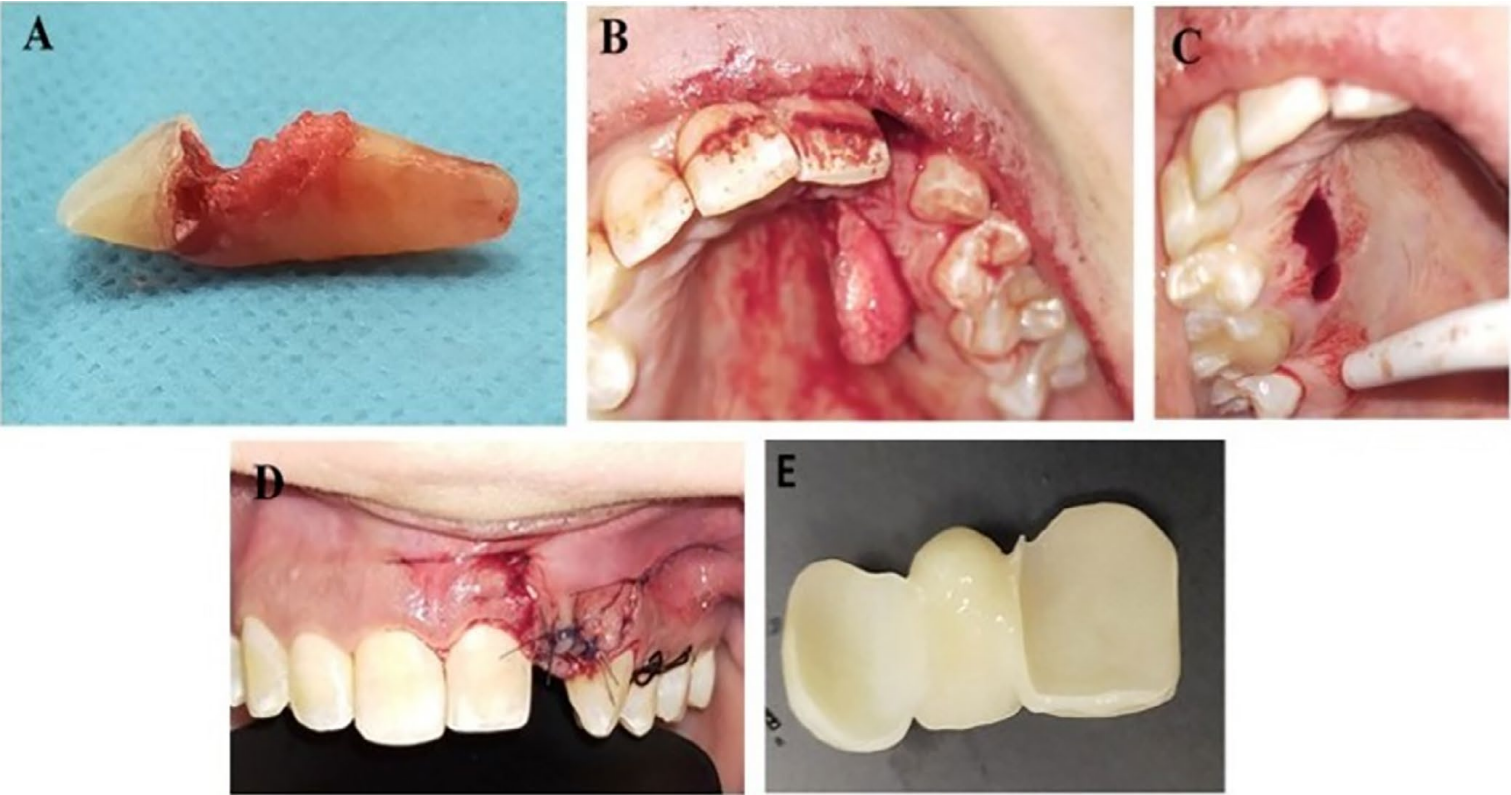
(A) Tooth after extraction. (B) Papilla‐preserving flap elevation. (C) Connective tissue punches from the contralateral palate for vertical soft tissue augmentation. (D) Application of autologous leukocyte platelet‐rich fibrin (L‐PRF) to the donor site suturing. (E) A temporary resin‐bonded bridge to maintain space and provide esthetic restoration.

The patient underwent thorough scaling, followed by intraoral digital scanning to design a temporary restoration. The tooth was extracted (Figure 3A). Labial preparations for veneers were performed before extraction on the maxillary central and canine teeth to provide abutment support for temporary veneer.

A papillary preservation flap was elevated to maintain interdental soft tissue integrity, and atraumatic extraction of the maxillary left lateral incisor was performed (Figure 3B). After debridement and removal of soft tissue inside the socket, socket preservation was achieved using a natural coral granule bone graft (Novocor Plus, B. & B. Dental S.r.l., Bologna, Italy), selected for its stability, osteoconductive properties, and reduced resorption. At the donor site, a rotational pedicle connective tissue flap was harvested to preserve keratinized palatal tissue and vascular supply, minimizing the risk of necrosis. Two additional connective tissue punches were harvested from the contralateral palate to augment vertical tissue height (Figure 3C). Autologous Leukocyte Platelet Rich Fibrin (L‐PRF) was applied to the donor site and palatal punches to stabilize the clot, accelerate healing, and reduce postoperative discomfort (Figure 3D). The temporary resin‐bonded bridge was immediately cemented to maintain space and provide an esthetic interim solution (Figure 3E). Photobiostimulation therapy (low‐level laser) was applied to enhance mitochondrial ATP production, improve microcirculation, promote tissue regeneration, and reduce inflammation and postoperative discomfort.

## 4 Follow‐Up and Outcomes

**FIGURE 4:**
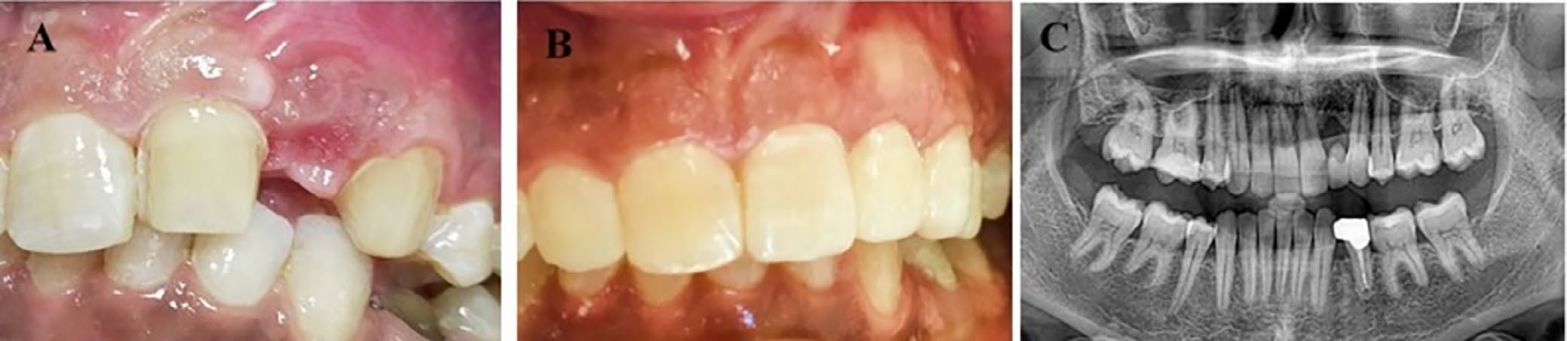
Clinical photographs after 5‐month (A) and 5‐year follow up (B). OPG radiograph after 5‐year follow‐up (C).

Healing was uneventful, with no signs of infection, tissue necrosis, or graft exposure at either the recipient or donor sites. The patient reported only mild postoperative discomfort, which resolved within the first week. At the 1‐month follow‐up, soft tissue healing was satisfactory, with good color match and integration of the grafted tissue. By 5 months, the augmented ridge demonstrated stable vertical soft tissue gain, maturation of the gingival margins, and well‐maintained keratinized tissue (Figure 4A). The provisional resin‐bonded bridge preserved the pontic space and supported the augmented tissues throughout the healing period. The temporary restoration was maintained until 6 months, after which the permanent resin‐bonded bridge was cemented, achieving ideal soft tissue and the soft tissue exhibited ideal length, thickness, and esthetic contours.

After 5 years follow up vertical soft tissue augmentation was successfully achieved, and the patient left with a stable, functional, and esthetic definitive restoration (Figure 4B,C).

This case highlights the effectiveness of a comprehensive, multidisciplinary approach in managing challenging esthetic situations in dentistry. Through the combination of surgical techniques such as socket preservation, vertical 3D soft tissue augmentation, and the use of a provisional resin‐bonded bridge, a stable, functional, and esthetically pleasing outcome was achieved. The integration of evidence‐based practices and interdisciplinary collaboration was essential in managing the distinctive challenges presented by this case.

## 5 Discussion

This case highlights the complexity of managing a severely resorbed and ectopically positioned maxillary lateral incisor, where conventional treatment approaches such as implant placement or orthodontic extrusion were not feasible due to anatomical limitations, root resorption, and aesthetic concerns. A comprehensive approach was adopted, including atraumatic extraction, socket preservation with a coral‐based graft, vertical soft tissue augmentation using a rotational pedicle flap with connective tissue punches, L‐PRF application, and photobiostimulation therapy to enhance healing. The provisional resin‐bonded bridge played a key role in maintaining the pontic space, supporting soft tissue maturation, and preserving papillary architecture and dental emergency profile. At 6 months, the patient demonstrated stable vertical soft tissue gain, favorable emergence profile, preservation of interdental papillae, uneventful healing, and a functionally and aesthetically harmonious permanent restoration.

Vertical 3D soft tissue augmentation is essential for achieving ideal esthetic and functional outcomes in pontic site development. Techniques such as the rotational pedicle flap and connective tissue grafts have proven effective in augmenting keratinized tissue width and mucosal thickness, thus improving the peri‐pontic soft tissue profile [9]. In this case, the combination of a rotational pedicle flap and connective tissue punches resulted in a significant gain in vertical soft tissue, which was maintained at the 6‐month follow‐up.

Socket preservation is essential to minimize alveolar ridge resorption following tooth extraction. The use of coral‐based grafts has been observed to provide stable volume maintenance and support new bone formation due to their osteoconductive properties [10, 11]. In this case, the natural coral granule bone graft preferred for socket preservation contributed to the stability of the ridge and facilitated subsequent soft tissue augmentation procedures [12]. The provisional resin‐bonded bridge fulfilled several functions: it maintained the pontic space, offered an esthetic interim solution, and supported the augmented tissues within the healing period. This approach aligns with current recommendations for managing esthetic complications around natural teeth, emphasizing the significance of conserving the interdental papilla and maintaining the natural gingival architecture [13, 14].

Along with surgical and restorative procedures, adjunctive photobiostimulation therapy (PBST) was utilized to promote healing. Low‐level laser therapy has been shown to stimulate mitochondrial ATP production, resulting in enhanced cellular metabolism and proliferation. Furthermore, PBST enhances local microcirculation, reduces postoperative inflammation, and promotes faster soft tissue regeneration, which is particularly beneficial in grafted or surgically manipulated sites. Several studies have demonstrated the positive effects of PBST in periodontal and oral surgery, reporting reduced postoperative pain, improved healing rate, and improved tissue quality [15, 16, 17]. In this case, the adjunctive use of PBST contributed to uneventful healing and improved patient comfort during the recovery phase.

In summary, the combined use of atraumatic extraction, socket preservation with a natural coral‐derived bone graft, and vertical soft tissue augmentation using a rotational palatal pedicle flap resulted in stable soft tissue architecture and satisfactory esthetic outcomes at long‐term follow‐up. The preservation of papillary height and pontic site contour was particularly noteworthy given the initial challenges posed by ectopic tooth position, severe cervical resorption, and limited mesiodistal space. The use of a vascularized pedicle flap, supplemented with connective tissue punches, allowed for predictable vertical tissue gain while minimizing donor site morbidity. Furthermore, restoration with a resin‐bonded prosthesis preserved the integrity of adjacent teeth and provided a conservative yet functional solution. Although this report represents a single clinical case, the favorable long‐term outcome suggests that this interdisciplinary approach may be a viable alternative for managing complex anterior maxillary defects where implant therapy is not feasible or desired. Future studies with larger sample sizes are warranted to further validate the predictability and long‐term stability of this technique.

## Author Contributions

**Mudar S. Kamal:** writing – review and editing, formal analysis. **Ghassan Habash:** investigation, writing – review and editing, formal analysis, writing – original draft. **Soher Nagi Jayash:** formal analysis, writing – review and editing. **Ayah Thikrallah:** investigation, writing – original draft, formal analysis.

## Funding

The authors would like to acknowledge the Biotechnology and Biological Sciences Research Council (BBSRC) for the Institute Strategic Programme Grant Funding BBS/E/RL/230001C, which provided salary support for SJ during the manuscript writing process.

## Consent

Written informed consent was obtained from the patient to publish this report in accordance with the journal's patient consent policy.

## Conflicts of Interest

The authors declare no conflicts of interest.

## Data Availability

The authors have nothing to report.

## References

[1] G. S. Silveira , N. V. de Almeida , D. M. T. Pereira , C. T. Mattos , and J. N. Mucha , “Prosthetic Replacement vs Space Closure for Maxillary Lateral Incisor Agenesis: A Systematic Review,” American Journal of Orthodontics and Dentofacial Orthopedics 150, no. 2 (2016): 228–237. 27476355 10.1016/j.ajodo.2016.01.018

[2] F. M. Spear , V. G. Kokich , and D. P. Mathews , “Interdisciplinary Management of Anterior Dental Esthetics,” Journal of the American Dental Association 137, no. 2 (2006): 160–169. 16521381 10.14219/jada.archive.2006.0140

[3] N. U. Zitzmann , G. Krastl , H. Hecker , C. Walter , T. Waltimo , and R. Weiger , “Strategic Considerations in Treatment Planning: Deciding When to Treat, Extract, or Replace a Questionable Tooth,” Journal of Prosthetic Dentistry 104, no. 2 (2010): 80–91. 20654764 10.1016/S0022-3913(10)60096-0

[4] A. Stumbras , P. Kuliesius , G. Januzis , and G. Juodzbalys , “Alveolar Ridge Preservation After Tooth Extraction Using Different Bone Graft Materials and Autologous Platelet Concentrates: A Systematic Review,” Journal of Oral & Maxillofacial Research 10, no. 1 (2019): e2. 10.5037/jomr.2019.10102 PMC6498816 31069040

[5] S. Said , “Extraction Site Management in the Esthetic Zone: Hard and Soft Tissue Reconstruction,” in Practical Advanced Periodontal Surgery (Wiley, 2020), 169–212.

[6] D. S. Thoma , G. I. Benić , M. Zwahlen , C. H. Hämmerle , and R. E. Jung , “A Systematic Review Assessing Soft Tissue Augmentation Techniques,” Clinical Oral Implants Research 20 (2009): 146–165. 19663961 10.1111/j.1600-0501.2009.01784.x

[7] M. Ehrenfeld and C.‐P. Cornelius , “Pedicled Flaps,” in Oral and Maxillofacial Surgery: Surgical Textbook and Atlas (Springer, 2023), 641–715.

[8] H. Salama , M. A. Salama , D. Garber , and P. Adar , “The Interproximal Height of Bone: A Guidepost to Esthetic Strategies and Soft Tissue Contours in Anterior Tooth Replacement,” Journal of Practical Periodontics and Aesthetic Dentistry 5 (2003): 64–73. 10093558

[9] G. Zucchelli , L. Tavelli , M. K. McGuire , et al., “Autogenous Soft Tissue Grafting for Periodontal and Peri‐Implant Plastic Surgical Reconstruction,” Journal of Periodontology 91, no. 1 (2020): 9–16. 31461778 10.1002/JPER.19-0350

[10] M. Schlee and M. Esposito , “Aesthetic and Patient Preference Using a Bone Substitute to Preserve Extraction Sockets Under Pontics. A Cross‐Sectional Survey,” European Journal of Oral Implantology 2, no. 3 (2009): 209–217. 20467631

[11] I. Rocchietta , L. Ferrantino , and M. Simion , “Vertical Ridge Augmentation in the Esthetic Zone,” Periodontology 2000 77, no. 1 (2018): 241–255. 29478252 10.1111/prd.12218

[12] E. El Chaar , S. Oshman , G. Cicero , et al., “Soft Tissue Closure of Grafted Extraction Sockets in the Anterior Maxilla: A Modified Palatal Pedicle Connective Tissue Flap Technique,” International Journal of Periodontics & Restorative Dentistry 37, no. 1 (2017): 99–107. 27977824 10.11607/prd.2746

[13] M. B. Blatz , T. Rotondo , S. Hant , and L. S. Prott , “Optimizing Hard and Soft‐Tissue Esthetics With Anterior Cantilever Zirconia Ceramic Resin‐Bonded Fixed Dental Prostheses,” Journal of Esthetic and Restorative Dentistry 38 (2026): 491–498. 40859853 10.1111/jerd.70025 PMC13062701

[14] A. Alrmali , S. Stuhr , M. H. Saleh , et al., “A Decision‐Making Tree for Evaluating an Esthetically Compromised Single Dental Implant,” Journal of Esthetic and Restorative Dentistry 35, no. 8 (2023): 1239–1248. 37449656 10.1111/jerd.13100

[15] S. Hosseinpour , J. Tunér , and R. Fekrazad , “Photobiomodulation in Oral Surgery: A Review,” Photobiomodulation, Photomedicine, and Laser Surgery 37, no. 12 (2019): 814–825. 31750798 10.1089/photob.2019.4712

[16] L. Gholami , S. Asefi , A. Hooshyarfard , et al., “Photobiomodulation in Periodontology and Implant Dentistry: Part 1,” Photobiomodulation, Photomedicine, and Laser Surgery 37, no. 12 (2019): 739–765. 31750783 10.1089/photob.2019.4710

[17] M. G. Newman , H. Takei , P. R. Klokkevold , and F. A. Carranza , Newman and Carranza's Clinical Periodontology E‐Book: Newman and Carranza's Clinical Periodontology E‐Book (Elsevier Health Sciences, 2018).

